# Reviewed article: Carvalho AF, Guaranha DDFK, Marmett B, Reis JM, Rosa BS, Souza CLE, Dalcin TC, Amantea SL, Machado FV. A multicenter study on the mental health of Brazilian adolescent mothers, 2024. Epidemiol Serv Saude. 2025:34;e20240226.

**DOI:** 10.1590/S2237-96222025v34e20240226.b

**Published:** 2025-09-08

**Authors:** Walter Ataalpa Freitas

**Affiliations:** 1 Universidade Estadual de Feira de Santana

**Table:** 

Rating	Excellent	Good	Average	Below average	Poor
Interest		✓			
Quality		✓			
Originality		✓			
Overall		✓			

**Table:** 

Questionnaire	Yes	No	Not applicable
Does the manuscript contain new and significant information to justify publication?	✓		
Does the Abstract (Summary) clearly and accurately describe the content of the article?		✓	
Is the problem significant and concisely stated?	✓		
Are the methods described comprehensively?		✓	
Are the interpretations and conclusions justified by the results?		✓	
Is adequate reference made to other work in the field?	✓		

## Manuscript Structure

Length of article is: Adequate

Number of tables is: Adequate

Number of figures is: Adequate

Please state any conflict(s) of interest that you have in relation to the review of this paper (state “none” if this is not applicable): None

## Comments

No manuscrito.

